# Supplementary material for: Negative refraction of light in an atomic medium
Source: Nat Commun. 2025 Feb 12;16:1433. doi: 10.1038/s41467-025-56250-w (PMC11822078; doi:10.1038/s41467-025-56250-w)
Supplement: Supplementary file 1 — Supplementary Information [file 41467_2025_56250_MOESM1_ESM.pdf]

# Supplementary Information: Negative Refraction of Light in an Atomic Medium

L. Ruks,[1,2,3] K. E. Ballantine,[4] and J. Ruostekoski[4]

[1] *NTT Basic Research Laboratories, NTT Corporation, 3-1 Morinosato Wakamiya, Atsugi, Kanagawa, 243-0198, Japan*

[2] *NTT Research Center for Theoretical Quantum Information, NTT Corporation, 3-1 Morinosato Wakamiya, Atsugi, Kanagawa, 243-0198, Japan*

[3] *Quantum Systems Unit, Okinawa Institute of Science and Technology Graduate University, Onna-son, Okinawa 904-0495, Japan*

[4] *Department of Physics, Lancaster University, Lancaster, LA1 4YB, United Kingdom*

(Dated: December 4, 2024)

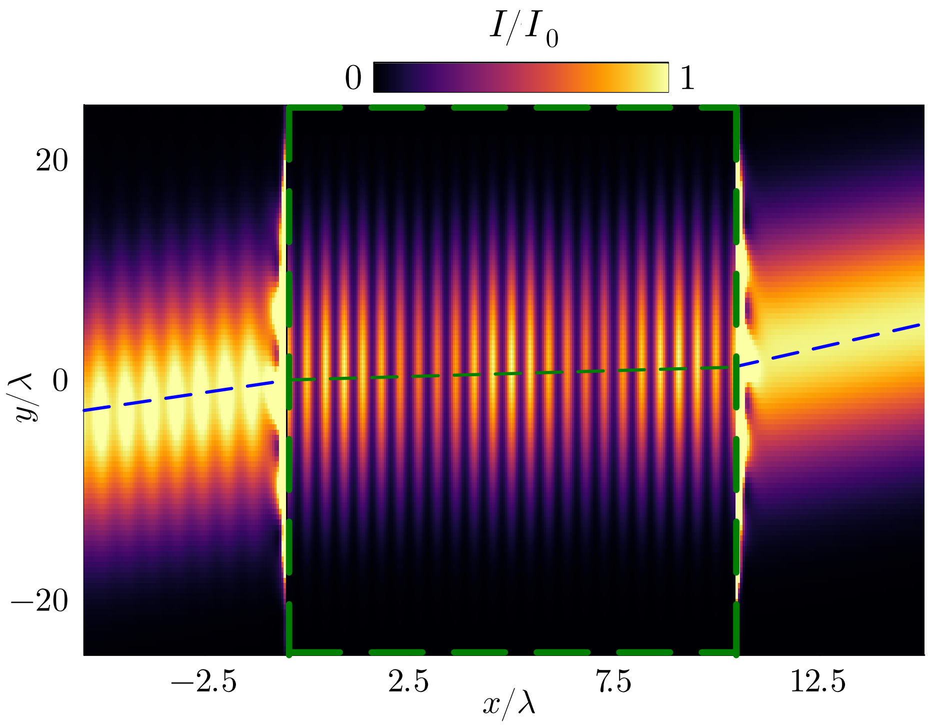


**Supplementary Fig. 1**. **Standard positive refraction and transmission of light through an atomic medium.** Refraction through a 25-layer atomic lattice observed in the normalised light intensity profile $I/I_0$ (outside the medium) and atomic polarisation density $|\langle\hat{\mathbf{P}}^+\rangle|^2/|\langle\hat{\mathbf{P}}^+\rangle|^2_{\mathrm{max}}$ (within the lattice delimited by the dashed green box) at plane $z = 0$, scaled by resonance wavelength $\lambda$, where $I_0 = 2\epsilon_0 c \max_{\mathbf{r}} |\boldsymbol{\mathcal{E}}^+(\mathbf{r})|^2$ represents the maximum incident intensity. For visualisation, $|\langle\hat{\mathbf{P}}^+\rangle|^2/|\langle\hat{\mathbf{P}}^+\rangle|^2_{\mathrm{max}}$ for point-like atoms is smoothed by convolution with a Gaussian of the root-mean-square widths $\sigma_x = 0.25a$ and $\sigma_y = 0.5a$. The blue dashed lines trace the peak light intensity, while the connecting green line marks the effective trajectory in the medium. All parameters are identical to Fig. 1(b) of the main text, except for the laser detuning, $\Delta = 1.08\gamma$, from atomic resonance. To obtain the observed standard positive refraction, the upper region of the transmission band, in Figs. 1(c) and (d) of the Main Text, is targeted. This corresponds to exciting polarisation eigenmodes (Main Text, Fig. 3(a)) featuring positive group velocities, $v_{g,y}^{(j)} = -\partial\delta^{(j)}(k_y, 0)/\partial k_y > 0$, so that the beam is displaced in the positive $y$-direction (Main Text, Fig. 3(c)) upon exiting the medium. This is in contrast to the negative refraction observed in Fig. 1(b) of the Main Text, where the lower region of the transmission band, featuring eigenmodes with negative in-plane group velocities, is targeted.

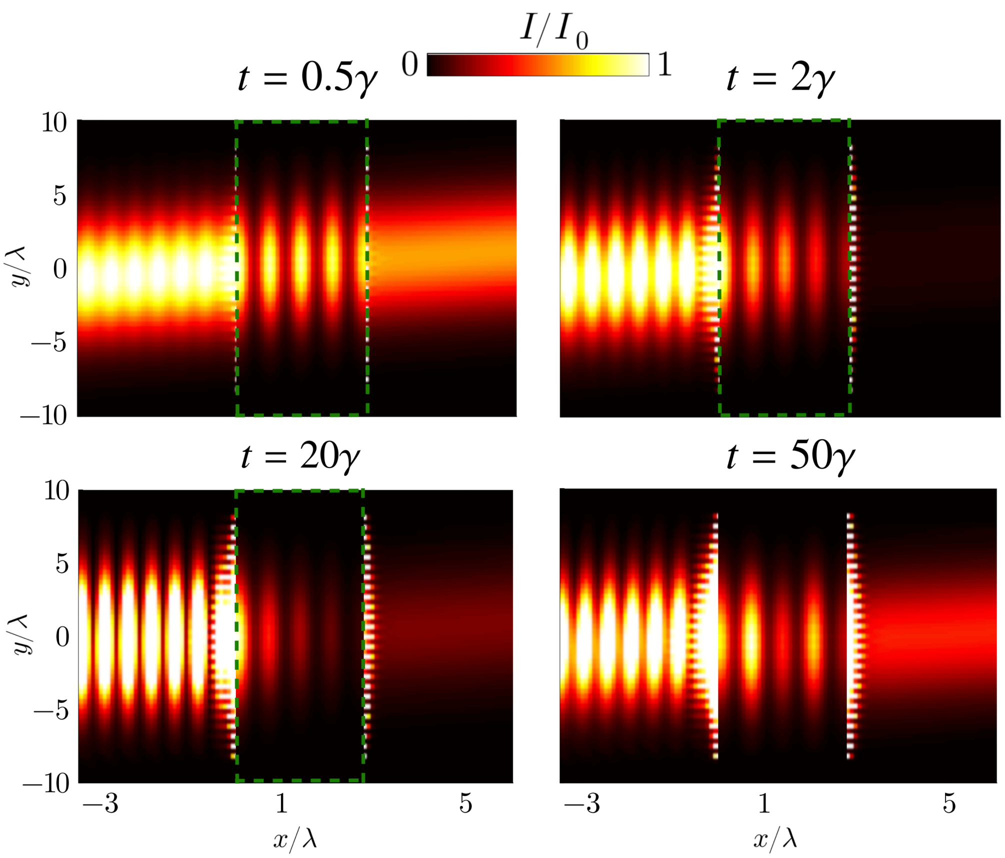


**Supplementary Fig. 2**. **Dynamics towards steady-state negative refraction.** Snapshots of the normalised light intensity profile (outside the medium) and smoothed atomic polarisation density within the atomic layers, as described in Supplementary Fig. 1, when the light is instantaneously switched on at $t = 0$. The atomic lattice and beam configuration otherwise identical to Fig. 5(a) of the Main Text. The system evolves according to Eq. (S1.7). In each snapshot, the polarisation density is normalised to its maximal value at that time.

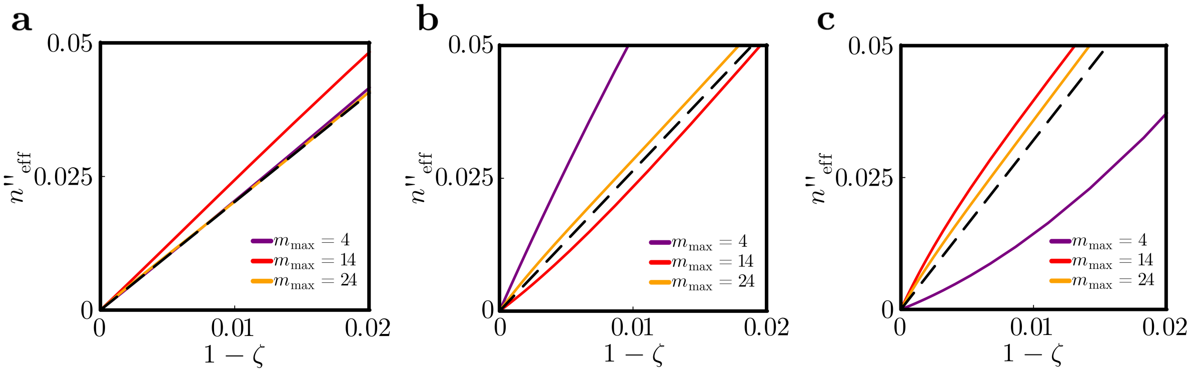


**Supplementary Fig. 3**. **Estimates of the imaginary part of the effective refractive index using mean-field approach.** Imaginary part $n''_{\text{eff}}$ of the effective group refractive index of for varying layer number $N_x$ and lattice imperfection parameter $\zeta$ of the phenomenological model. The beam is incident with a detuning $\Delta = 0.63\gamma$ and angle (a) $\theta = 0.25 \times \pi$ (b) $\theta = 0.2 \times \pi$ (c) $\theta = 0.17 \times \pi$, whilst all other parameters are as in Fig. 3(a) of the Main Text. We estimate the imaginary part of the effective refractive index based on the beam attenuation, according to the formula $n''_{\text{eff}} = 1/(kL)$, where $L = -a(N_x - 1)/[\ln(T_\zeta/T_0)]$ is the attenuation length and $T_\zeta$ is the power transmission observed for $\zeta$ (solid lines). The dashed line denotes the approximation $L \simeq -\nabla_\perp\delta/[(1-\zeta)\gamma]$, obtained using the out-of-plane group velocity $-\nabla_\perp\delta = -\partial\delta/\partial k_x > 0$ for the $x$-component of the wavevector $k_x$ of the phase-matched resonant ($\Delta = -\delta$) Bloch wave excitation in the fully infinite lattice ($N_x = \infty$).

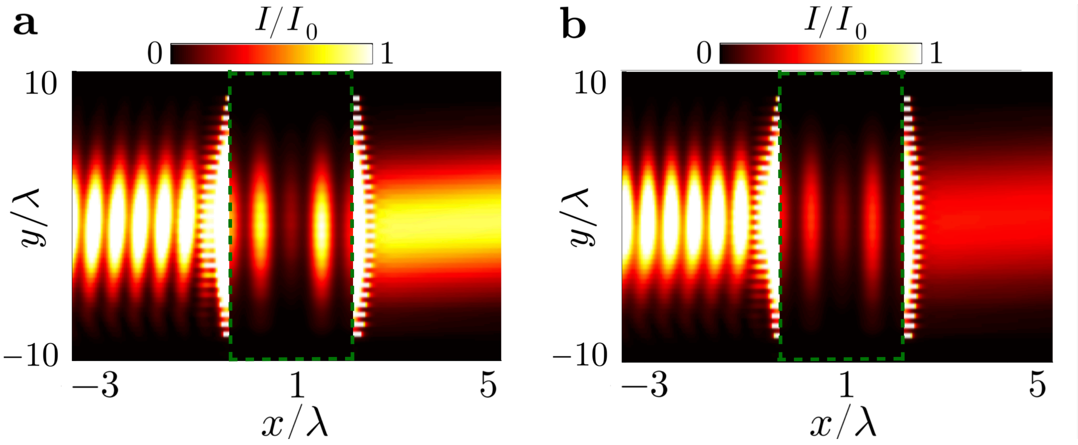


**Supplementary Fig. 4**. **Negative refraction of light beyond the low light intensity limit.** The normalised light intensity profile (outside the medium) and smoothed atomic polarisation density within the atomic layers, as described in Supplementary Fig. 1, through a cubic $5\times25\times25$ array of atoms (a) in the low-light-intensity limit and (b) beyond the low-light-intensity limit, with the maximum of the Rabi frequency for a Gaussian beam at the origin $|\mathcal{R}^{+}(\mathbf{0})|/\gamma \simeq 0.025$. All parameters are otherwise identical to Fig. 5(a) of the Main Text, with (a) reproduced here for direct comparison with (b). In (b), we solve for the steady-state of nonlinearly responding atoms in a semi-classical approximation [1], retaining the excited-state populations.

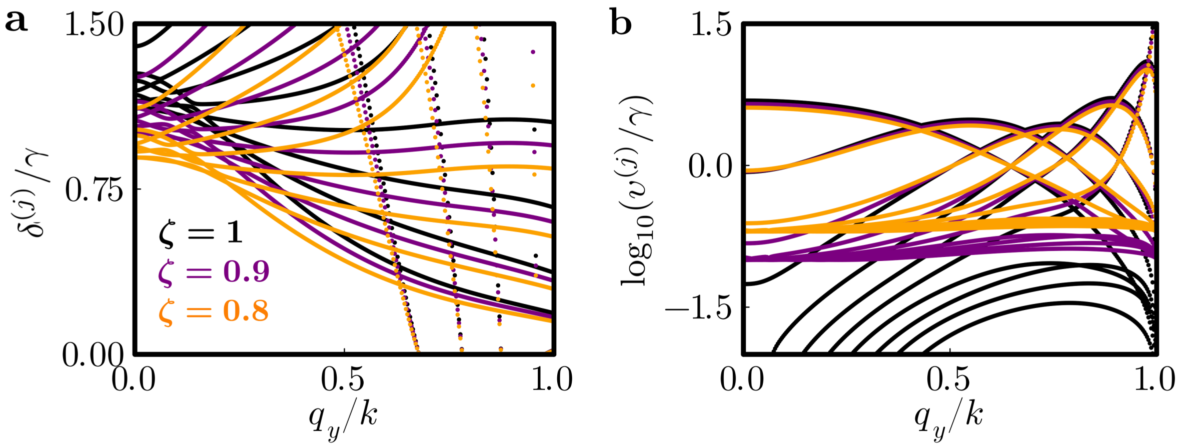


**Supplementary Fig. 5**. **Variation of band structure with lattice imperfections in the phenomenological model.** (a) Line shifts and (b) log-linewidths of collective resonances of the atomic lattice for the lattice imperfection parameter $\zeta = 0.8, 0.9, 1.0$ in the phenomenological model. Here, agreement has separately been confirmed with the band structure obtained using momentum summation. Lattice parameters otherwise correspond to those of the five-layer system in Fig. 3(b) of the Main Text.

## Supplementary Note 1. Light Interacting with Atoms

### A. General Formalism

In this note, we briefly recap the general theory of atoms interacting with light that is employed for the calculation of light propagation in an atomic array. The general theory is presented for a $J = 0 \rightarrow J' = 1$ degenerate transition, with the frequency $\omega_0$, although in the numerics we also consider two-level atoms. The positive frequency component of the atomic polarisation density operator $\hat{\mathbf{P}}^+(\mathbf{r})$ consists of the sum over the atomic dipoles

$$\hat{\mathbf{P}}^+(\mathbf{r}) = \sum_{j\nu} \delta(\mathbf{r} - \mathbf{r}_j)\mathbf{d}_{ge}^{(\nu)}\hat{\sigma}_{j\nu}^-. \tag{S1.1}$$

Here, $\hat{\sigma}_{j\nu}^+ = (\hat{\sigma}_{j\nu}^-)^\dagger = |e_{j\nu}\rangle\langle g_j|$ is the raising operator to the excited state $\nu = \pm 1, 0$ for atom $j$, with the dipole matrix element $\mathbf{d}_{ge}^{(\nu)} = \mathcal{D}\hat{\mathbf{e}}_\nu$, $[\mathbf{d}_{ge}^{(\nu)}]^* = \mathbf{d}_{eg}^{(\nu)}$ given in terms of the reduced dipole matrix element $\mathcal{D}$ and the circular polarisation unit vectors $\hat{\mathbf{e}}_\pm = \mp\frac{1}{\sqrt{2}}(\hat{\mathbf{e}}_x \pm i\hat{\mathbf{e}}_y)$, $\hat{\mathbf{e}}_0 = \hat{\mathbf{e}}_z$. We consider stationary atoms and write the atomic operators and light fields as slowly varying amplitudes by factoring out the rapid oscillations at the laser frequency $\omega$, such as $\exp(-i\omega t)$ from $\hat{\sigma}_{j\nu}^-$. In the length gauge, obtained by the Power-Zienau-Woolley transformation [2–4], the interaction between the atoms and light is then given in the rotating wave approximation by

$$\hat{H}_{\mathrm{al}} = -\sum_{j\nu} \hbar\Delta\hat{\sigma}_{j\nu}^{ee} - \frac{1}{\epsilon_0}\int d^3\mathbf{r}\left(\hat{\mathbf{D}}^+(\mathbf{r})\cdot\hat{\mathbf{P}}^-(\mathbf{r}) + \mathrm{H.c.}\right), \tag{S1.2}$$

for the positive-frequency component $\hat{\mathbf{D}}^+(\mathbf{r})$ of the displacement field and laser detuning $\Delta = \omega - \omega_0$, whilst $\hat{\sigma}_{j\nu}^{ee} = \hat{\sigma}_{j\nu}^+\hat{\sigma}_{j\nu}^-$ represents the excited population of level $\nu$ on atom $j$. The Hamiltonian contains a self-polarisation term that can be neglected in our system of non-overlapping point atoms [4]. In the following, we analyze the light propagation in the limit of low light intensity to first order in the incident light field amplitude [3] and write the positive frequency component of the atomic polarisation amplitude as the expectation value of the atomic lowering operators $\mathcal{D}\boldsymbol{\mathcal{P}}^{(j)} = \sum_\nu \mathbf{d}_{ge}^{(\nu)}\langle\hat{\sigma}_{j\nu}^-\rangle$. The atoms are illuminated by a coherent laser field with the positive frequency component $\boldsymbol{\mathcal{E}}^+(\mathbf{r})$ and polarisation $\hat{\mathbf{e}}_{\mathrm{in}}$

$$\boldsymbol{\mathcal{E}}^+(\mathbf{r}) = \hat{\mathbf{e}}_{\mathrm{in}}\mathcal{E}\, g(\mathbf{r})\, e^{i\mathbf{k}\cdot\mathbf{r}}, \tag{S1.3}$$

for the dominant wavevector $\mathbf{k}$. We examine the cases where the incident field is a plane wave, for $g(\mathbf{r}) = 1$, or a Gaussian beam in the paraxial approximation, with the familiar Gaussian focusing along the plane $\mathbf{r} - \hat{\mathbf{k}}(\hat{\mathbf{k}}\cdot\mathbf{r})$ perpendicular to the propagation direction $\hat{\mathbf{k}} = \mathbf{k}/k$ and the beam divergence along a cone of constant $\hat{\mathbf{k}}\cdot\hat{\mathbf{r}}$ [5]. For the broad beams with $k_z = 0$ considered in the Main Text, confinement may be assumed in the $xy$ plane only for simplicity, with a negligible impact on calculations. The positive frequency component of the total field $\hat{\mathbf{E}}^+(\mathbf{r}) = \boldsymbol{\mathcal{E}}^+(\mathbf{r}) + \hat{\mathbf{E}}_s^+(\mathbf{r})$ is then the sum of $\boldsymbol{\mathcal{E}}^+(\mathbf{r})$ and the corresponding scattered field amplitude, $\hat{\mathbf{E}}_s^+$,

$$\epsilon_0\langle\hat{\mathbf{E}}_s^+(\mathbf{r})\rangle = \sum_j \mathsf{G}(\mathbf{r} - \mathbf{r}_j)\mathcal{D}\boldsymbol{\mathcal{P}}^{(j)}, \tag{S1.4}$$

where $\mathsf{G}_{\nu\mu}(\mathbf{r}) = \hat{\mathbf{e}}_\nu^*\cdot\mathsf{G}(\mathbf{r})\hat{\mathbf{e}}_\mu$ is the dipole radiation kernel (or Green's function)

$$\mathsf{G}_{\nu\mu}(\mathbf{r}) = \left[\frac{\partial}{\partial r_\nu}\frac{\partial}{\partial r_\mu} - \delta_{\nu\mu}\boldsymbol{\nabla}^2\right]\frac{e^{ikr}}{4\pi r} - \delta_{\nu\mu}\delta(\mathbf{r}). \tag{S1.5}$$

This can be expanded for a dipole $\mathbf{d}$ at the origin [6]

$$\mathsf{G}(\mathbf{r})\mathbf{d} = -\frac{\mathbf{d}\delta(\mathbf{r})}{3} + \frac{k^3}{4\pi}\left\{(\hat{\mathbf{r}}\times\mathbf{d})\times\hat{\mathbf{r}}\frac{e^{ikr}}{kr}\,[3\hat{\mathbf{r}}\,(\hat{\mathbf{r}}\cdot\mathbf{d}) - \mathbf{d}]\left[\frac{i}{(kr)^2} - \frac{1}{(kr)^3}\right]e^{ikr}\right\}, \tag{S1.6}$$

where $\hat{\mathbf{r}} = \mathbf{r}/|\mathbf{r}|$. The atomic dipole amplitude of the atom $j$ is driven by the incident field Rabi frequency $\mathcal{R}^+(\mathbf{r}_j) = \mathcal{D}\mathcal{E}^+(\mathbf{r}_j)/\hbar$ and the field scattered by all the other atoms, and satisfies the differential equation

$$\frac{d\boldsymbol{\mathcal{P}}^{(j)}}{dt} = (i\Delta - \gamma)\boldsymbol{\mathcal{P}}^{(j)} + i\boldsymbol{\mathcal{R}}^+(\mathbf{r}_j) + i\xi\sum_{\ell\neq j}\mathsf{G}(\mathbf{r}_j - \mathbf{r}_\ell)\boldsymbol{\mathcal{P}}^{(\ell)}. \tag{S1.7}$$

Here $\gamma$, the single-atom linewidth, and $\xi$ are given by

$$\gamma = \frac{\mathcal{D}^2 k^3}{6\pi\hbar\epsilon_0}, \quad \xi = \frac{6\pi\gamma}{k^3}, \tag{S1.8}$$

whilst the single-atom resonance shifts are absorbed in the detuning $\Delta$. The dynamics can be cast in the form

$$\dot{\mathbf{b}} = i(\mathcal{H} + \delta\mathcal{H})\mathbf{b} + \mathbf{f}, \tag{S1.9}$$

where (with $\nu, \mu = \pm 1, 0$) $\mathbf{b}_{3j-1+\nu} = \mathcal{P}^{(j)}_\nu$, $\mathbf{f}_{3j-1+\nu} = i\hat{\mathbf{e}}^*_\nu \cdot \mathcal{R}^+(\mathbf{r}_j)$, and the diagonal elements of $\mathcal{H}$ are $i\gamma$. The diagonal matrix $\delta\mathcal{H}$ contains $\Delta$. The non-diagonal part consists of the light-mediated coupling

$$\mathcal{H}_{3j-1+\nu, 3\ell-1+\mu} = \xi \mathsf{G}_{\nu\mu}(\mathbf{r}_j - \mathbf{r}_\ell), \quad j \neq \ell. \tag{S1.10}$$

The collective excitation eigenmodes are the eigenvectors of $\mathcal{H}$ and the corresponding eigenvalues $\delta_j + i\upsilon_j$ consist of the collective linewidths $\upsilon_j$ and line shifts $\delta_j = \omega_0 - \omega_j$ from the single-atom resonance. The steady-state solution of Eq. (S1.7) represents the standard multiple scattering relation for the self-consistently induced dipoles

$$\mathcal{D}\boldsymbol{\mathcal{P}}^{(j)} = \alpha\epsilon_0 \boldsymbol{\mathcal{E}}^+(\mathbf{r}_j) + \alpha \sum_{\ell \neq j} \mathsf{G}(\mathbf{r}_j - \mathbf{r}_\ell)\mathcal{D}\boldsymbol{\mathcal{P}}^{(\ell)}, \tag{S1.11}$$

where the atomic polarisability

$$\alpha = -\frac{\mathcal{D}^2}{\hbar\epsilon_0(\Delta + i\gamma)} = -\frac{\xi}{\Delta + i\gamma}. \tag{S1.12}$$

### B. Optical Response of Stacked Arrays of Infinite In-plane Extent

Here, we detail the theory employed to calculate the optical response of layered planar arrays of infinite transverse extent. In the demonstration of negative refraction of light, we consider a cubic 3D Bravais lattice of $N_x \times N_y \times N_z$ atoms. This can be described as a set of 2D square arrays parallel to the $yz$ plane, with lattice vectors $(0, a, 0)$, $(0, 0, a)$, and a unit cell of area $\mathcal{A}' = a^2$. The 2D layers are stacked along the $x$-axis at the positions $x_\ell = \ell a$ ($\ell = 0, \ldots, N_x - 1$). Individual atomic positions are then denoted by $\mathbf{r}_\mathbf{n} = \ell a \hat{\mathbf{e}}_x + \mathbf{r}_{\parallel j}$ with $\mathbf{n} = (\ell, j)$ specifying the layer $\ell$ and the in-plane position $\mathbf{r}_{\parallel j}$, respectively.

The stationary-state optical response can in principle be directly calculated from the linear set of equations (S1.11) for the atoms and Eq. (S1.4) for light. However, problems can arise when dealing with an infinitely extending array due to the slow convergence of real-space summation and the potential for mathematical issues [7] stemming from the lack of absolute convergence in these sums. On the other hand, a computationally manageable approach for large ensembles is offered by the momentum-space representations in the case of sufficiently large 2D layers where the lattice edge effects can be neglected by considering each layer as translationally invariant. This is obtained by removing high-frequency modes (always an implicit assumption in the non-relativistic electrodynamics) by introducing an isotropic cutoff term in the momentum summations [1]. The momentum $\mathbf{q}$ cutoff term $\exp[-q^2\eta^2/4]$, for the cutoff length $\eta$, can also physically represent the Gaussian $\exp[-r^2/\eta^2]$ smearing of atomic positions in each lattice site due to quantum or thermal fluctuations [7]. The optical response for the atoms at fixed positions is obtained by taking the limiting value for the cutoff parameter $\eta \to 0$ in the end of the calculation, while the non-zero $\eta$ approximately describes finite-depth optical lattice potentials. In the following, we formulate the case where each of the layers extends infinitely in the $y$ and $z$ directions, allowing for compact momentum-space representations of light propagation.

We consider the incident field of Eq. (S1.3), with the dominant wavevector $\mathbf{k} = (k_x, \mathbf{k}_\parallel)$ for the translationally invariant layers in the $yz$ plane, where $g(\mathbf{r}) \neq 1$ in Eq. (S1.3) introduces a finite width for the distribution of wavevectors. The atomic polarisation amplitudes are represented by Bloch waves $\boldsymbol{\mathcal{P}}^{(\ell,j)} = \sum_{\mathbf{q}_\parallel} \boldsymbol{\mathcal{P}}_\ell(\mathbf{q}_\parallel) e^{i\mathbf{q}_\parallel \cdot \mathbf{r}_{\parallel j}}$ for the in-plane wavevectors $\mathbf{q}_\parallel$ excited by light. Substituting the Bloch waves into Eqs. (S1.4) and (S1.11) yields for the scattered light

$$\epsilon_0 \langle \hat{\mathbf{E}}^+_s(\mathbf{r}) \rangle = \sum_{\mathbf{q}_\parallel} \sum_{\ell=0}^{N_x-1} \mathsf{G}^{\mathrm{L}}(x - x_\ell, \mathbf{r}_\parallel; \mathbf{q}_\parallel)\mathcal{D}\boldsymbol{\mathcal{P}}_\ell(\mathbf{q}_\parallel), \tag{S1.13}$$

and the atomic polarisation amplitudes of the 2D layers of atoms [compare with Eq. (S1.7)]

$$\dot{\boldsymbol{\mathcal{P}}}_\ell(\mathbf{q}_\parallel) = (i\Delta - \gamma)\boldsymbol{\mathcal{P}}_\ell(\mathbf{q}_\parallel) + i\boldsymbol{\mathcal{R}}^+_\ell(\mathbf{q}_\parallel) + i\xi \sum_{m=0}^{N_x-1} \mathsf{G}^{\mathrm{L}}(x_\ell - x_m, \mathbf{0}; \mathbf{q}_\parallel)\boldsymbol{\mathcal{P}}_m(\mathbf{q}_\parallel), \tag{S1.14}$$

expressed now via Bloch wave excitations of each of the layers. Here we have defined the layer propagator $\mathsf{G}^{\mathrm{L}}$ in terms of the resulting lattice sums,

$$\mathsf{G}^{\mathrm{L}}(x,\mathbf{r}_{\|};\mathbf{q}_{\|}) = \sum_j e^{i\mathbf{q}_{\|}\cdot\mathbf{r}_{\|j}}\mathsf{G}(\mathbf{r}_{\|}-\mathbf{r}_{\|j}+x\hat{\mathbf{e}}_x), \quad x\neq 0, \tag{S1.15}$$

$$\mathsf{G}^{\mathrm{L}}(0,\mathbf{0};\mathbf{q}_{\|}) = \sum_{j\neq 0} e^{i\mathbf{q}_{\|}\cdot\mathbf{r}_{\|j}}\mathsf{G}(\mathbf{r}_{\|j}), \tag{S1.16}$$

where in the second expression we have excluded the $j = 0$ term $\mathbf{r}_{\|0} = \mathbf{0}$. Since the 2D layers are translationally invariant, this choice of reference atom is arbitrary. Whilst Eq. (S1.15) describes interlayer interactions, the term (S1.16) is needed to describe the self-interaction of each 2D layer and is present even in the case of a single layer only.

The collective excitation eigenmodes of Eq. (S1.10) can be expressed in terms of the layer propagators. In an infinite system, they form a continuous band structure. From Eq. (S1.14), we obtain a linear system in $\mathbf{q}_{\|}$-space analogously to Eq. (S1.9)

$$\dot{\mathbf{b}}(\mathbf{q}_{\|}) = i[\mathcal{H}(\mathbf{q}_{\|})+\delta\mathcal{H}]\mathbf{b}(\mathbf{q}_{\|})+\mathbf{f}(\mathbf{q}_{\|}), \tag{S1.17}$$

where $\mathcal{H}$ (and $\delta\mathcal{H}$) is a $3N_x\times 3N_x$ matrix

$$\mathcal{H}_{3n-1+\nu,3m-1+\mu}(\mathbf{q}_{\|}) = \xi\mathsf{G}^{\mathrm{L}}_{\nu\mu}(x_n-x_m,\mathbf{0};\mathbf{q}_{\|}) + i\gamma\delta_{\nu\mu}\delta_{nm}, \tag{S1.18}$$

$\mathbf{b}_{3m-1+\nu}(\mathbf{q}_{\|}) = \mathcal{P}_{m\nu}(\mathbf{q}_{\|})$, and $\mathbf{f}_{3j-1+\nu}(\mathbf{q}_{\|}) = i\hat{\mathbf{e}}^*_\nu\cdot\mathcal{R}^+(\mathbf{q}_{\|})$. We use Eqs. (S1.15) and (S1.16) in Eq. (S1.18) to diagonalise $\mathcal{H}(\mathbf{q}_{\|})$ and obtain the eigenvalues $\delta^{(j)}+i\upsilon^{(j)}$ that represent the collective line shifts $\delta^{(j)}=\delta^{(j)}(\mathbf{q}_{\|})$ and linewidths $\upsilon^{(j)}=\upsilon^{(j)}(\mathbf{q}_{\|})$ of each collective excitation eigenmode in band $j$.

For a single atomic layer, the induced amplitude $\mathcal{P}_0(\mathbf{q}_{\|})$ of the layer satisfies [1, 8, 9], for an eigenmode,

$$\begin{aligned}\dot{\mathcal{P}}_0(\mathbf{q}_{\|}) &= (i\Delta-\gamma)\mathcal{P}_0(\mathbf{q}_{\|}) + i\mathcal{R}^+_0(\mathbf{q}_{\|}) + i\xi\mathsf{G}^{\mathrm{L}}(\mathbf{0},\mathbf{0};\mathbf{q}_{\|})\mathcal{P}_0(\mathbf{q}_{\|})\\ &= [i\Delta+i\delta_s(\mathbf{q}_{\|})-\gamma-\tilde{\gamma}_s(\mathbf{q}_{\|})]\mathcal{P}_0(\mathbf{q}_{\|}) + i\mathcal{R}^+_0(\mathbf{q}_{\|}),\end{aligned} \tag{S1.19}$$

where $\delta_s(\mathbf{q}_{\|})$ and $\tilde{\gamma}_s(\mathbf{q}_{\|})$ are the real and imaginary parts of $\xi\mathsf{G}^{\mathrm{L}}(\mathbf{0},\mathbf{0};\mathbf{q}_{\|})$, representing the collective line shift $\delta_s(\mathbf{q}_{\|})$ and linewidth $\upsilon_s(\mathbf{q}_{\|}) = \gamma+\tilde{\gamma}_s(\mathbf{q}_{\|})$ of the single-layer eigenmode.

The two lattice sums (S1.15) and (S1.16) are different but we evaluate both of them in momentum space. We begin by expanding the spherical scattered wave near the planar array as plane waves using the Weyl identity [9–13]. Expressing Eq. (S1.5) as

$$\mathsf{G}_{\nu\mu}(\mathbf{r}) = \frac{i}{8\pi^2}\int d^2\mathbf{q}_{\|}\left(\frac{\partial}{\partial r_\nu}\frac{\partial}{\partial r_\mu}+\delta_{\nu\mu}k^2\right)\frac{1}{k_\perp}e^{i\mathbf{q}_{\|}\cdot\mathbf{r}_{\|}}e^{ik_\perp|x|}, \tag{S1.20}$$

where $k_\perp = (k^2-q_{\|}^2)^{1/2}$, we introduce the Fourier transform of the radiation kernel on the array plane

$$\mathsf{G}(\mathbf{r}) = \int\frac{d^2\mathbf{q}_{\|}}{(2\pi)^2}e^{i\mathbf{q}_{\|}\cdot\mathbf{r}_{\|}}\tilde{\mathsf{G}}^{\|}(\mathbf{q}_{\|}), \tag{S1.21}$$

where $\tilde{\mathsf{G}}^{\|}(\mathbf{q}_{\|})$ may be read off upon comparison with Eq. (S1.20). We substitute Eq. (S1.21) into Eq. (S1.15) and use the (2D) Poisson summation formula

$$\sum_j e^{i\mathbf{k}_{\|}\cdot\mathbf{r}_{\|j}} = \frac{(2\pi)^2}{\mathcal{A}'}\sum_j\delta^{(2)}(\mathbf{k}_{\|}-\mathbf{g}_j), \tag{S1.22}$$

for reciprocal-lattice vectors $\mathbf{g}_j$ of the 2D in-plane lattice (such that $e^{i\mathbf{g}_j\cdot\mathbf{r}_{\|\ell}}=1$). In the case $(x,\mathbf{r}_{\|})\neq(0,\mathbf{0})$ (or $(0,\mathbf{r}_{\|j})$ by periodicity) we then perform the $\mathbf{q}_{\|}$-space integral to obtain

$$\begin{aligned}\mathsf{G}^{\mathrm{L}}_{\nu\mu}(x,\mathbf{r}_{\|};\mathbf{k}_{\|}) &= \frac{1}{\mathcal{A}'}\sum_j\tilde{\mathsf{G}}^{\|}_{\nu\mu}(\mathbf{k}_{\|}+\mathbf{g}_j)e^{i(\mathbf{k}_{\|}+\mathbf{g}_j)\cdot\mathbf{r}_{\|}}\\ &= \frac{i}{2\mathcal{A}'}\sum_j\frac{1}{k_\perp(\mathbf{g}_j)}\left(-k^{\mathrm{B}}_{j\nu}k^{\mathrm{B}}_{j\mu}+\delta_{\nu\mu}k^2\right)e^{i(\mathbf{k}_{\|}+\mathbf{g}_j)\cdot\mathbf{r}_{\|}}e^{ik_\perp(\mathbf{g}_j)|x|}\\ &= \frac{ik^2}{2\mathcal{A}'}\sum_j\frac{1}{k_\perp(\mathbf{g}_j)}\mathsf{P}_{\perp\nu\mu}(\mathbf{k}^{\mathrm{B}}_j)e^{i(\mathbf{k}_{\|}+\mathbf{g}_j)\cdot\mathbf{r}_{\|}}e^{ik_\perp(\mathbf{g}_j)|x|},\end{aligned} \tag{S1.23}$$

where we have now incorporated $\mathbf{g}_j$ via

$$k_\perp(\mathbf{g}_j) = \sqrt{k^2 - (k_y + g_{jy})^2 - (k_z + g_{jz})^2}, \tag{S1.24}$$

with the $j$-th Bragg order wavevector $\mathbf{k}_j^{\mathrm{B}} = [\mathrm{sgn}(x)k_\perp(\mathbf{g}_j), \mathbf{k}_\parallel + \mathbf{g}_j]$, and where $\mathsf{P}_\perp(\mathbf{k})$ denotes the projector onto the subspace orthogonal to $\mathbf{k}$. The self-lattice interaction term for $(x, \mathbf{r}_\parallel) = (0, \mathbf{0})$ is obtained using the Poisson summation formula (S1.22)

$$\mathsf{G}^{\mathrm{L}}(0, \mathbf{0}; \mathbf{k}_\parallel) = \frac{1}{\mathcal{A}'} \sum_j \tilde{\mathsf{G}}^{\parallel}(\mathbf{k}_\parallel + \mathbf{g}_j) - \mathsf{G}(0). \tag{S1.25}$$

To assign this sum a meaningful physical value, we use the aforementioned high-momentum cutoff term $\exp[-q^2\eta^2/4]$ that regularises the summation [7, 14, 15]. The explicit expression

$$\tilde{\mathsf{G}}^{\parallel *}_{\nu\mu}(\mathbf{q}_\parallel) = \int \frac{dq_\perp}{2\pi k^2} \frac{k^2\delta_{\nu\mu} - q_\nu q_\mu}{k^2 - (q_\parallel^2 + q_\perp^2) + i\epsilon} e^{-q^2\eta^2/4} e^{iq_\perp x}, \tag{S1.26}$$

is obtained by introducing the 3D momentum-space Fourier transform $\mathsf{G}(\mathbf{q})$ of the radiation kernel:

$$\mathsf{G}(\mathbf{r}) = \int \frac{d^3\mathbf{q}}{(2\pi)^3} e^{i\mathbf{q}\cdot\mathbf{r}} \tilde{\mathsf{G}}(\mathbf{q}), \quad \tilde{\mathsf{G}}(\mathbf{q}) = \frac{1}{k^2} \frac{q_\mu q_\nu - k^2\delta_{\mu\nu}}{k^2 - q^2 + i\epsilon}. \tag{S1.27}$$

The momentum-space representation offers a computationally manageable approach when solving the linear system (S1.14) for the excitations of each layer $\mathcal{P}_\ell$, even for hundreds of transversely infinite layers. After self-consistently calculating the atomic polarisation amplitudes for the 2D layers $\mathcal{P}_\ell(\mathbf{q}_\parallel)$, the coherently scattered field can be obtained from Eq. (S1.13).

The power transmission $T$ of light through the sample is calculated, with the expectation values taken over fluctuating atomic positions,

$$T = \frac{\int \sum_\mu \langle \hat{\mathbf{e}}_\mu \cdot \hat{\mathbf{E}}^+(\mathbf{r})\hat{\mathbf{E}}^-(\mathbf{r}) \cdot \hat{\mathbf{e}}_\mu^* \rangle d\Omega}{\int \boldsymbol{\mathcal{E}}^+(\mathbf{r}) \cdot \boldsymbol{\mathcal{E}}^-(\mathbf{r}) d\Omega}, \tag{S1.28}$$

where the summation is over the orthogonal polarisations and the integration is performed over a solid angle $d\Omega$, capturing light that propagates to the positive $x$-direction, sufficiently far away from the atoms to exclude the evanescent field components. To analyse focussed transmission, which is also valid for incoherent scattering with fluctuating atomic positions, we consider a small collection surface in the plane $x = a(N_x - 1) + 2\lambda$, extending across $-10\lambda \leq y, z \leq 10\lambda$.


[1] J. Ruostekoski, Cooperative quantum-optical planar arrays of atoms, Phys. Rev. A **108**, 030101 (2023).
[2] C. Cohen-Tannoudji, J. Dupont-Roc, and G. Grynberg, *Photons and Atoms: Introduction to Quantum Electrodynamics* (John Wiley & Sons, New York, 1989).
[3] J. Ruostekoski and J. Javanainen, Quantum field theory of cooperative atom response: Low light intensity, Phys. Rev. A **55**, 513 (1997).
[4] M. D. Lee, S. D. Jenkins, and J. Ruostekoski, Stochastic methods for light propagation and recurrent scattering in saturated and nonsaturated atomic ensembles, Phys. Rev. A **93**, 063803 (2016).
[5] M. Born and E. Wolf, *Principles of Optics*, 7th ed. (Cambridge University Press, Cambridge, UK, 1999).
[6] J. D. Jackson, *Classical Electrodynamics*, 3rd ed. (Wiley, New York, 1999).
[7] M. Antezza and Y. Castin, Spectrum of Light in a Quantum Fluctuating Periodic Structure, Phys. Rev. Lett. **103**, 123903 (2009).
[8] J. Javanainen and R. Rajapakse, Light propagation in systems involving two-dimensional atomic lattices, Phys. Rev. A **100**, 013616 (2019).
[9] E. Shahmoon, D. S. Wild, M. D. Lukin, and S. F. Yelin, Cooperative resonances in light scattering from two-dimensional atomic arrays, Phys. Rev. Lett. **118**, 113601 (2017).
[10] H. Benisty, J.-J. Greffet, and P. Lalanne, *Introduction to Nanophotonics*, 1st ed. (Oxford University Press, Oxford, 2022).
[11] L. Novotny and B. Hecht, *Principles of Nano-Optics*, 2nd ed. (Cambridge University Press, Cambridge, 2012).
[12] P. A. Belov and C. R. Simovski, Homogenization of electromagnetic crystals formed by uniaxial resonant scatterers, Phys. Rev. E **72**, 026615 (2005).
[13] P. A. Belov and C. R. Simovski, Boundary conditions for interfaces of electromagnetic crystals and the generalized ewald-oseen extinction principle, Phys. Rev. B **73**, 045102 (2006).
[14] M. Antezza and Y. Castin, Fano-hopfield model and photonic band gaps for an arbitrary atomic lattice, Phys. Rev. A **80**, 013816 (2009).
[15] J. Perczel, J. Borregaard, D. E. Chang, H. Pichler, S. F. Yelin, P. Zoller, and M. D. Lukin, Photonic band structure of two-dimensional atomic lattices, Phys. Rev. A **96**, 063801 (2017).
